# Unveiling tissue-specific transcriptional adaptations in iPSC-derived fibroblasts via co-culture systems

**DOI:** 10.1186/s13287-025-04537-6

**Published:** 2025-07-30

**Authors:** Amar J. Azad, Alessandro Bentivogli, Henrike Germar, Dana Wörz, Elena Lizunova, Max J. Cumberland, January Weiner, Sarah Hedtrich

**Affiliations:** 1 Käthe-Beutler-Haus, Berlin Institute of Health at Charité, Berlin, Germany; 2 Centre for Heart Research, The Westmead Institute for Medical Research, Westmead, NSW Australia; 3 School of Biomedical Engineering, The University of British Columbia, 2350 Health Sciences Mall, Vancouver, BC V6T 1Z3 Canada; 4 Faculty of Pharmaceutical Sciences, The University of British Columbia, Vancouver, BC Canada; 5 Centre for Blood Research & Life Science Institute, Life Sciences Centre, University of British Columbia, Vancouver, BC Canada

**Keywords:** iPSCs, Fibroblast heterogeneity, iPSC-derived fibroblasts, Single-cell deconvolution, Fibroblast plasticity, Organ-specific fibroblasts

## Abstract

**Background:**

Induced pluripotent stem cell-derived fibroblasts (iFBs) hold promise for autologous disease modelling, but their ability to replicate tissue-specific fibroblast characteristics remains unclear. Fibroblasts exhibit significant heterogeneity, with distinct subtypes playing critical roles in organ function and integrity. This study investigates whether iFBs can acquire tissue-specific transcriptional profiles through co-culture with cells from different germ layers, including skin (keratinocytes), heart (cardiomyocytes), gut (intestinal cells), and lung (bronchial epithelial cells).

**Methods:**

iFBs were co-cultured directly or indirectly with organ-specific cell types, followed by bulk RNA sequencing and pathway analysis. Transcriptional profiles were compared to primary fibroblasts using principal component analysis (PCA), large single-cell databases of over 20,000 cells for single-cell deconvolution and targeted qPCR validation. Statistical significance was assessed via one-way ANOVA.

**Results:**

Transcriptomic analysis revealed that iFBs exhibit transcriptional plasticity, adopting molecular phenotypes aligned with their co-culture environment across all germ layers. Paracrine signalling induced transient tissue-specific changes in indirectly co-cultured iFBs, but sustained interactions were required for stable adaptations. Pathway analysis highlighted functional shifts, such as TGF-β activation in cardiac iFBs and ECM remodelling in dermal iFBs. However, single-cell deconvolution showed incomplete tissue specification, with iFBs retaining mixed fibroblast subpopulations.

**Conclusions:**

These findings demonstrate that iFBs can adopt tissue-specific transcriptional profiles, supporting their potential for modelling fibrotic microenvironments in 3D in vitro systems. However, the partial and transient nature of these adaptations underscores the need to validate whether transcriptional changes translate to functional fibroblast behaviours, such as ECM dysregulation or aberrant TGF-β signalling, in complex tissue models. Optimising co-culture conditions to stabilise these phenotypes will be critical for leveraging iFBs in fibrosis research, drug screening, and personalised disease modelling.

**Supplementary Information:**

The online version contains supplementary material available at 10.1186/s13287-025-04537-6.

## Introduction

Human-based (disease) models, including organoids, bioengineered 3D tissue models and organ-on-chip setups, aim to bridge the gap between traditional 2D cell cultures, animal-based in vivo models and the physiological complexity of native human tissues and organs. As such, human-based models offer a controlled environment to investigate cell-cell interactions, extracellular matrix (ECM)– cell interactions, and tissue-specific functions while minimising interspecies-related differences inherent to animal studies. Achieving high levels of biomimicry requires the integration of all tissue-relevant cell types to accurately replicate the complex (patho) physiological interactions that occur in vivo [1].

Fibroblasts, which are present in all connective tissues, are crucial for maintaining structural integrity and homeostasis by synthesising and remodelling ECM and orchestrating cellular responses during injury repair, inflammation, and other stress-related processes [2]. Fibroblasts are highly heterogenous and exist in distinct subtypes contributing to crucial roles in organ integrity and cellular interactions [3]. This heterogeneity, in which fewer than 20% of fibroblast-enriched genes overlapped between four organs (heart, skeletal muscle, intestine, and bladder), reflects their diverse functions across organs, including structural support, fibrosis, immune modulation, and tissue repair [4]. Dermal fibroblasts for example contribute to skin homeostasis by participating in wound healing and producing different ECM components relative to cardiac fibroblasts which regulate myocardial structure and contribute to electrical conduction in the heart [5, 6].

Organ-specific fibroblasts play a crucial role in developing complex 2D and 3D disease models, particularly in the context of autologous human-based systems. However, the limited availability of primary fibroblasts from certain organs (e.g., the heart) has made induced pluripotent stem cell (iPSC)-derived fibroblasts (iFBs) an essential alternative. iFBs have been successfully applied in a wide range of contexts, both as standalone models and within co-culture systems [7, 8]. These cells offer distinct advantages for patient specific disease modelling, by ensuring donor-donor variability and allogeneic interactions do not confound experimental readouts. This is particularly important in immunological contexts, where the influence of genetic differences between donors can confound experimental outcomes [9].

However, the ability of iFBs to replicate the tissue-specific characteristics and functional plasticity of native fibroblasts remains underexplored. A key challenge has been the lack of specific markers to define fibroblast identity and function. A recent review of over 3,000 studies highlighted the difficulty in identifying specific fibroblast markers [10]. Despite advances in single-cell RNA sequencing, the absence of reliable markers makes it difficult to determine whether generated iFBs are universal precursors or organ-specific subtypes, limiting their application in complex disease models.

To address these challenges, this study investigates whether iFBs can acquire tissue-specific transcriptional profiles when co-cultured with cells from three germ layers: cardiomyocytes (mesoderm), keratinocytes (ectoderm), and bronchial and intestinal epithelial cells (endoderm). Using a direct co-culture setup, we analysed transcriptional changes in iFBs by bulk RNA-Seq, comparing them to independently cultured iFBs and assessing the impact of direct versus indirect co-culture, where cell contact is eliminated in the latter. Our findings demonstrate that iFBs exhibit transcriptional plasticity, responding dynamically to co-culture environments across all germ layers. This suggests their potential for integration into complex human-based models but highlights the necessity of sustained interaction for stable tissue-specific adaptations.

## Materials and methods

### Human tissue material and maintenance

Adult stem cell (ASC)-derived jejunal cells (intestinal cells) were expanded as organoids in EIF medium (Advanced DMEM/F12, Primocin™ at 100 µg/ml, GlutaMAX at 1x, HEPES at 10mM, Y-27632 at 10µM, B27 supplement at 1x, NAC at 1mM, EGF at 50ng/ml, Gastrin I at 10nM, TGF-β inhibitor (A83-01) at 0.5µM, WNT Surrogate-Fc Fusion Protein at 0.5nM, Rspo3-Fc Conditioned Medium at 1 µg/ml, Noggin at 50ng/ml, IGF-I at 100ng/ml, FGF-2 at 50 ng/ml, and Prostaglandin E2 at 1µM) and then seeded at 750 000 cells/cm^2^ onto transwells pre-coated with Matrigel diluted at a ratio of 1:50, to form monolayers for terminal differentiation.

Keratinocytes were cultivated in EpiLife (ThermoScientific, USA) supplemented with Human Keratinocyte Growth Supplement (ThermoScientific, USA) on untreated tissue-culture plastics at an initial cell density of 10 000 cells/cm^2^. Normal human bronchial epithelial cells (NHBE) and lung fibroblasts (NHLFb) were purchased from Epithelix (Switzerland). NHBEs were cultured in PneumaCult Ex Plus (STEMCELL Technologies, Canada) on untreated tissue-culture plastics at an initial cell density of 20 000 cells/cm^2^. Human intestinal fibroblasts were purchased from ScienCell (USA). Human cardiac fibroblasts (306v-05a, Cell Applications Inc) were kindly obtained as a gift from Prof. Sophie van Linthout (Berlin Institute of Health Center for Regenerative Therapies, Germany). All primary fibroblasts were cultured in Fibroblast growth medium (FGM: DMEM (Sigma, D6546) supplemented with 10% FBS, 2mM L-glutamine and 400U/ml Penicillin/Streptomycin) at an initial cell density of 5 000 cells/cm^2^.

### iPSC maintenance and iFB generation

iPSCs were cultured in E8 MDC-HomeBrew medium (Technology Platform Pluripotent Stem Cells of the Max Delbrück Center, Germany). Cells were passaged using TrypLE Express (Gibco, USA) and plated in culture medium supplemented with 1.2 µg/mL iMatrix-511 (NP892-011, Reprocell, Japan) for adhesion and 10µM Y-27,632 (1254, Tocris, UK). Medium was refreshed daily.

**Fig. 1:**
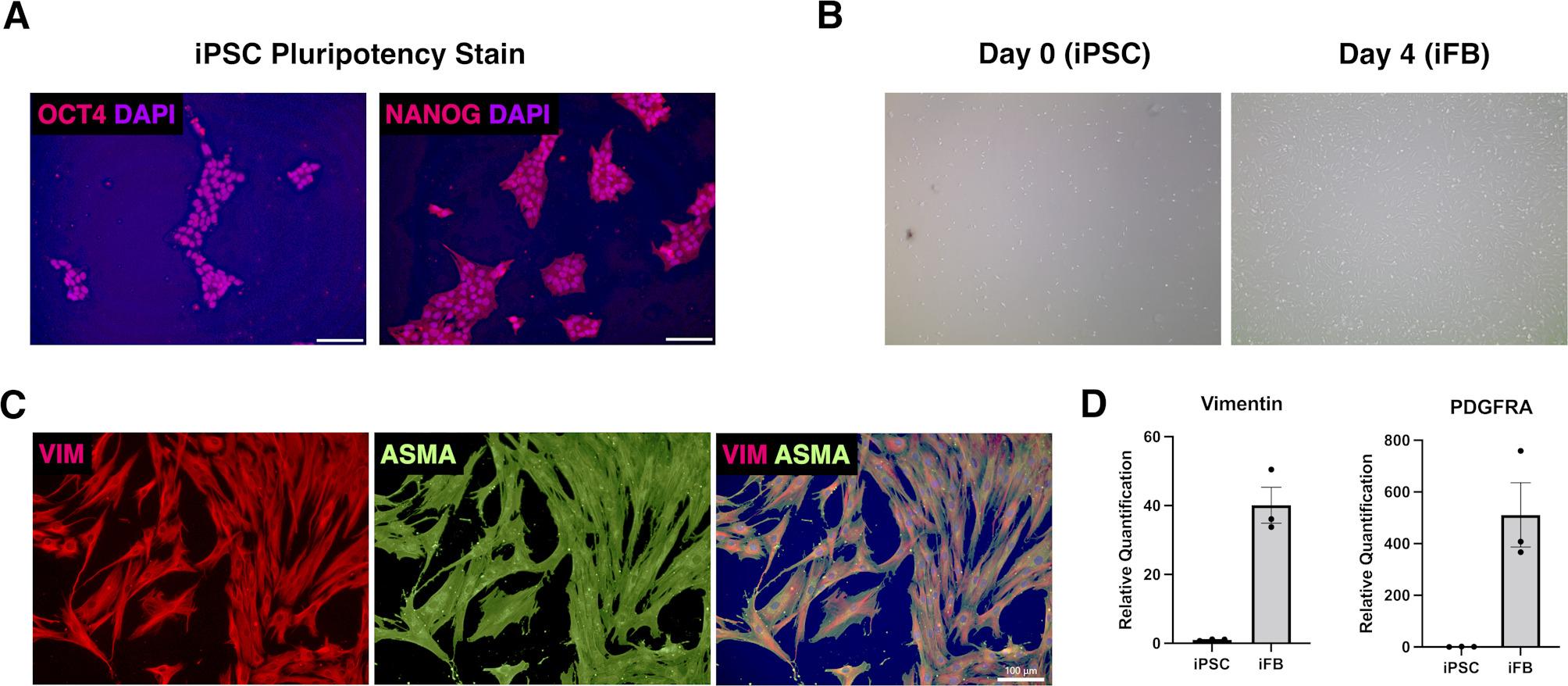
(**A**) Immunofluorescence staining against pluripotency markers OCT4 and NANOG expressed in iPSCs. (**B**) Brightfield progression of iPSC to iFB from day 0 to day 4. (**C**) Immunofluorescence staining against vimentin and alpha smooth actin (ASMA). (**D**) Relative quantification of vimentin and PDGFRA transcript expression in iPSC vs iFB. All data are presented as mean ± SEM

iPSCs were differentiated into iFBs by adapting a previously published protocol [11] after being seeded at 2 × 10^4^ cells/cm^2^ on iMatrix-511 in E8 medium with 10µM Y-27,632. The next day, differentiation began in CnT-PR-F (CellnTEC, Switzerland), 10% FBS, 25ng/mL BMP4, transitioning to Fibroblast Growth Medium (FGM) on Day 4. From Days 6–10, 10µM SB-43,125 was added to prevent myofibroblast transition. Basic iFB characterisation is found in Fig. 1.

### Generation of iPSC-derived cardiomyocytes

At ~ 90% iPSC confluence, cardiac differentiation was initiated in RPMI-1640 + B27 (1:50) (without insulin) (Gibco, USA) with CHIR99021 (Tocris, UK) (8µM Day 0; 4µM Day 1), followed by 5µM IWR-1 (Tocris, UK) on Day 3. On Day 7, cells were switched to RPMI-1640 + B27 (with insulin). Spontaneous contractions typically began around Days 8–10. All media from Days 0–7 contained 30µM L-ascorbic acid (Sigma-Aldrich, USA).

### Co-culture of iFBs setup

#### Preparation of iFBs

On day 10 post-differentiation initiation, iFBs were passaged using 0.25% trypsin-EDTA for 5 min at 37 °C. Following detachment, cells were collected in FGM, counted using a hemocytometer, and resuspended in culture medium. For co-culture experiments, iFBs were seeded at 3 000 cells/cm^2^ onto the Matrigel-coated basal side of inverted transwell inserts (Corning, USA). Cells were allowed to adhere and settle for 45 min at 37 °C in a humidified incubator with 5% CO₂. The inserts were then placed into standard culture wells containing FGM supplemented with 10µM Y-27,632 for submerged culture. Cells were maintained under these conditions for 24 h prior to the introduction of organ-specific cell types.

#### Direct co-culture setup

The following day, keratinocytes, NHBEs, iPSC-derived cardiomyocytes, or ASC-derived jejunal cells (Fig. S1) were seeded on the apical side of the same transwell insert at an appropriate cell density for each cell type. This configuration allowed cells to be placed in close proximity on opposite sides of the same porous membrane, allowing localised signalling, thereby constituting a direct co-culture system. Direct co-cultures were maintained for 7 days in respective media (RPMI-1640 + B27 with insulin for cardiac, EpiLife for dermal, PneumaCult ExPlus for pulmonary, EIF for intestinal). iFBs were then collected for analysis; control iFBs were maintained separately but in the same media for direct comparison.

#### Indirect co-culture setup

For indirect co-culture, iFBs were seeded on a Matrigel-coated plate at a cell density of 3 × 10^4^ cm^2^; a transwell with organ-specific cells was placed above. After 7 days, some co-cultures were harvested, while others had the insert removed and were cultured for another 7 days to assess the persistence of co-culture effects.

### Harvesting of cells

After the specified culture period, iFBs were harvested by gentle scraping. The cells from multiple wells were pooled, washed with PBS, and processed immediately or stored at − 80 °C for subsequent analyses. All experiments were performed in triplicate to ensure reproducibility.

### RNA quantification

The quantity and quality of the RNA samples were assessed using the following methods. Preliminary quality control was performed on 1% agarose gel electrophoresis to test RNA degradation and potential contamination. Sample purity and preliminary quantitation were measured using Bioanalyzer 2100 (Agilent Technologies, USA) and it was also used to check the RNA integrity and final quantitation.

### Library construction, quality control and sequencing of the library

For library preparation, we used the Novogene NGS RNA Library Prep Set (PT042). The mRNA present in the total RNA sample was isolated with magnetic beads of oligos d(T)25 (Vazyme, China). This method is known as polyA-tailed mRNA enrichment. Subsequently, mRNA was randomly fragmented and cDNA synthesis proceeds using random hexamers and the reverse transcriptase enzyme. Once the synthesis of the first chain was finished, the second chain was synthesised with the addition of an Illumina buffer (non-directional library preparation). With this and together with the presence of dNTPs, RNase H and polymerase I from E. Coli, the second chain was obtained by Nick translation. Resulting products underwent purification, end-repair, A-tailing and adapter ligation. Fragments of the appropriate size were enriched by PCR, where indexed P5 (AATGATACGGCGACCACCGAGA (5’-3’)) and P7 (CGTATGCCGTCTTCTGCTTG-P7’ (5’-3’)) primers were introduced with final products purified.

The library was checked with Qubit 2.0 (Invitrogen, USA) and real-time PCR (Thermo Fisher Scientific, USA) for quantification and bioanalyzer Agilent 2100 for size distribution detection. Quantified libraries were pooled and sequenced on the Illumina Novaseq X platform, according to effective library concentration and data amount using the paired-end 150 strategy (PE150).

### RNA-seq and principal component analysis

Reads were mapped against the human genome v. GRCh38 (http://www.ncbi.nlm.nih.gov/projects/genome/assembly/grc/human/), p7 using the STAR aligner [12] v. 2.7.3a (https://github.com/alexdobin/star/releases). Gene counts were obtained through featureCounts program [13] v. 2.0.3 (http://subread.sourceforge.net). Counts were log-normalised using the rlog function from the DESeq2 package [14] v. 1.38 (https://bioconductor.org/packages/DESeq2/). Principal components were calculated using the R function prcomp, and Pearson correlation coefficients were calculated between the samples using the principal components.

### Pathway enrichment analysis

Selected KEGG pathways were retrieved using the keggList() and keggGet() functions from the KEGGREST R package. Gene symbols were extracted and standardised to uppercase. Ensembl gene IDs from the expression matrix were mapped to HGNC symbols using the biomaRt package (*hsapiens_gene_ensembl* dataset). Differential expression results were obtained from a custom pipeline built with the Rseasnap package (https://github.com/bihealth/Rseasnap). For each group comparison, genes with|log₂ fold change| ≥ 1 and adjusted *p*-value (padj) ≤ 0.05 were selected. For each pathway, the top 10 DEGs were identified and visualised in heatmaps using the pheatmap package.

### Single-cell semiprofiler

Single-cell deconvolution of bulk RNAseq data was done as according to Wang et al. [15]. This approach allowed us to estimate deeply the composition of cell types within complex tissue samples by leveraging reference single-cell transcriptomic data. As our reference single-cell transcriptomic data, we utilised the comprehensive dataset from the Tabula Sapiens project, which provides a high-resolution single-cell transcriptomic atlas across multiple human tissues [15].

To tailor this reference dataset to our specific analysis, we applied filtering criteria to isolate and retain only cells annotated as fibroblasts. Additional filtering was applied, selecting up to 2,000 cells per subtype with at least 1,000 cells, and removed low-quality cells (expressing < 200 genes or genes detected in < 5 cells) to construct a refined fibroblast-specific reference matrix. As we lacked single-cell RNA-seq data from our own samples, this curated dataset served as a biologically relevant reference under the assumption that primary fibroblast subtype diversity is broadly representative of our iFB populations (Fig. S4).

The resulting fibroblast-specific single-cell profiles were integrated into the scSemiProfiler model to estimate the relative abundance and heterogeneity of fibroblast populations within our iFB bulk RNA-seq samples.

### Targeted gene expression via RT-qPCR

For targeted gene expression analysis, markers were selected due to their expression in organ-resident fibroblasts. For example, *POSTN* and *TBX20* mark cardiac fibroblasts involved in ECM remodelling and heart development; *KRT14* and *MMP2* are indicative of dermal identity; *NPNT* and *HHIP* reflect pulmonary fibroblast functions linked to lung matrix interactions; and *BMP4*, *FBLN1*, and *CXCL12* serve as markers for intestinal fibroblasts, with *CXCL12* reflecting broader fibroblast activity.

Total RNA was extracted from iFBs. Briefly, models were lysed in PureLink RNA Mini Kit lysis buffer (Invitrogen, USA). RNA was isolated according to the manufacturer’s protocol. Total RNA was quantified, and cDNA was synthesised, using iScript™ cDNA Synthesis Kit (Bio-Rad Laboratories, USA). The subsequent RT-qPCR was performed using SYBR Green I Master Mix (Bio-Rad Laboratories, USA). 18 S served as a housekeeping gene control. Primer sequences are listed in Supplemental Table 1.

### Statistical analysis

Statistical analysis was performed with RStudio Version 2024.09.1 + 394 and GraphPad Prism 10 (Version 10.4.1) software. Values are expressed as means ± SEM from at least three biological replicates. A one-way ANOVA was used for the comparison of > two parametric groups. *P* <.05 were considered statistically significant.

## Results

### iPSC-derived fibroblasts exhibit mesenchymal features and loss of pluripotency

To confirm the generation of fibroblast-like cells from iPSCs, we performed morphological and molecular characterisation across early stages of differentiation. Immunofluorescence staining confirmed the expression of canonical pluripotency markers *OCT4* and *NANOG* in undifferentiated iPSCs, indicating a robust starting pluripotent population (Fig. 1A). Upon initiation of differentiation, iPSCs underwent marked morphological changes between day 0 and day 4, adopting an elongated, spindle-shaped morphology characteristic of mesenchymal cells (Fig. 1B).

Consistent with a mesenchymal transition, immunofluorescence analysis at day 10 revealed high expression of vimentin and α-smooth muscle actin (ASMA), both markers associated with fibroblast identity and cytoskeletal reorganisation (Fig. 1C). At the transcriptional level, qPCR analysis demonstrated a significant upregulation of *vimentin* and *PDGFRA*, two key fibroblast-associated genes, in iFBs compared to undifferentiated iPSCs (Fig. 1D). These data confirm successful downregulation of pluripotency and acquisition of fibroblast-like characteristics prior to co-culture.

### Co-cultured iFB RNAseq analysis

**Fig. 2:**
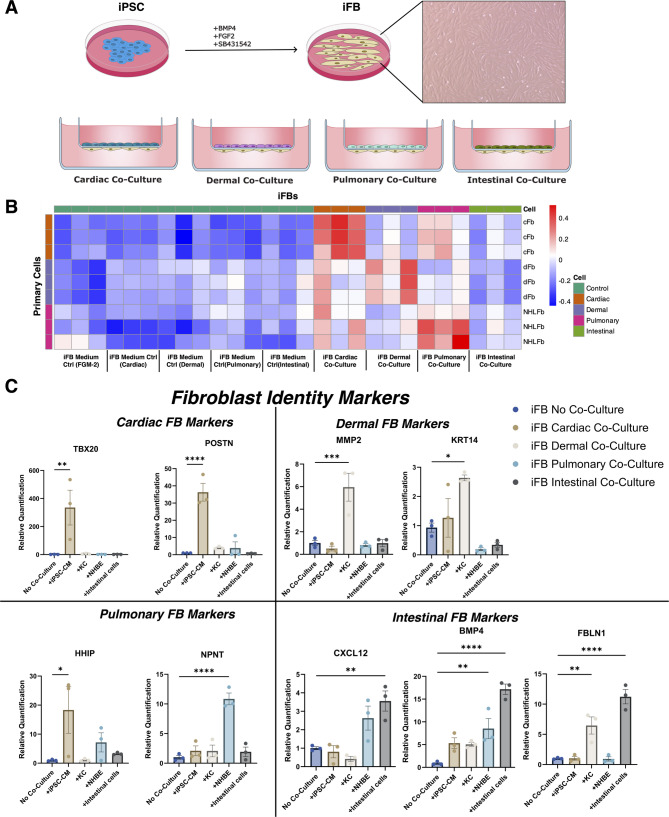
(**A**) Schematic representation illustrating culture conditions leading to iFB differentiation, followed by co-culture configurations for cardiac, dermal, pulmonary, and intestinal cells on the apical side of the transwell with fibroblasts seeded on the underside of the transwell. (**B**) PCA correlation heatmap comparing RNA-seq datasets of iFBs with cardiac (cFB), dermal (dFB), and normal human lung fibroblasts (NHLFs), and intestinal fibroblasts demonstrating tissue-specific transcriptional adaptations. (**C**) Expression of organ-specific fibroblast markers across all four iFB-co-cultures relative to independently cultured iFBs in fibroblast maintenance medium, FGM, measured via qPCR. All data are presented as mean ± SEM, *n* = 3 per cell line per condition. One-way ANOVA was performed, *P <.05, **P <.01, ***P <.001, P****<0.0001

To assess transcriptional responses of iFBs in different microenvironments, iFBs were co-cultured with human keratinocytes (KCs), bronchial epithelial cells (NHBE), myocardial cells (iPSC-CMs), and intestinal cells (Fig. 2A). Each co-culture was maintained in cell-specific media, with corresponding medium-only controls. Following co-culture, iFBs were subjected to bulk RNA-sequencing. PCA-based correlation and expression heatmaps (Fig. 2B, Fig. S4) revealed clustering of co-cultured iFBs with primary fibroblasts from their respective dermal, cardiac, and pulmonary tissues, indicating transcriptional alignment and similarity. This clustering was consistent across three independent iPSC lines.

To validate transcriptional adaptations, we performed a head-to-head comparison of co-cultured iFBs with tissue-derived fibroblasts, selecting key markers based on their known relevance to specific organ environments. Targeted qPCR analysis (Fig. 2C) showed varying degrees of organ specificity, consistent with Fig. 2B and Fig. S3A. *POSTN*, *MMP2*, and *NPNT* were preferentially upregulated in cardiac, dermal, and pulmonary iFB co-cultures, respectively. In contrast, *HHIP* and *KRT14* were broadly induced across multiple co-culture conditions. As a representative sample, *HHIP* was tested in both primary cardiac and lung fibroblasts, with no significant difference in gene expression found between them (Fig. S3B). *BMP4* and *FBLN1* exhibited strong induction in intestinal co-cultures but were also significantly elevated in other contexts. For example, *FBLN1* was also upregulated in dermal conditions, and *BMP4* showed increased expression across all co-culture conditions compared to controls (Fig. 2C).

### Pathway enrichment analysis

**Fig. 3:**
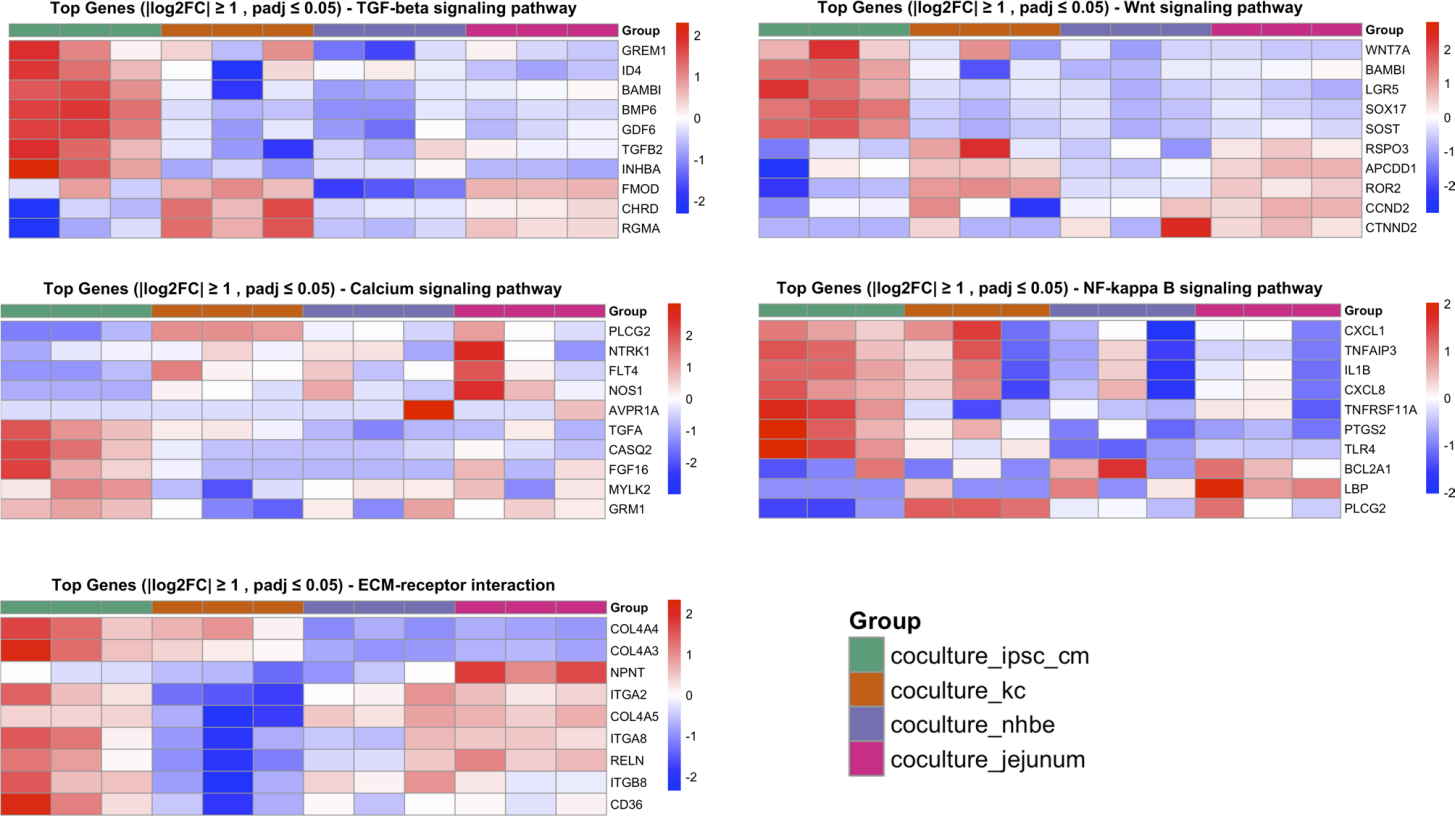
Heatmaps depicting differentially expressed (DE) genes across key KEGG pathways in iFBs. Groups include iFBs co-cultured with iPSC-derived cardiomyocytes (coculture_ipsc_cm), keratinocytes (coculture_kc), NHBEs (coculture_nhbe), and ASC-derived jejunal cells (coculture_jejunum) compared to the expression of independently cultured iFBs in the respective basal medium of co-culture e.g. EpiLife, PneumaCult ExPlus

Pathway analysis of differentially expressed genes revealed that iFBs respond to co-culture conditions with distinct transcriptional adaptations (Fig. 3, Fig. S3A), though overall pathway enrichment was modest across the pathways examined. Compared to iFBs maintained in tissue-specific basal medium alone, co-cultured iFBs exhibited significant alterations in key signalling pathways associated with tissue-specific fibroblast function.

In cardiac co-cultures, genes within the TGF-β signalling pathway were significantly upregulated. Calcium signalling components were also elevated. Wnt signalling showed strong context-specific modulation: cardiac iFBs upregulated *WNT7A*, *LGR5*, and *SOX17*, while dermal iFBs exhibited a general downregulation of these Wnt related genes.

The ECM receptor interaction pathway was notably enriched in dermal iFBs, with a diverse expression profile suggesting robust ECM remodelling activity. In contrast, pulmonary iFBs, while clustering near primary lung fibroblasts in global transcriptional space (Fig. 2, Fig. S4), exhibited limited activation of canonical pathways associated with lung fibroblast identity.

Distinct pathway activation patterns were also observed between intestinal and pulmonary iFBs, despite their shared endodermal origin. PI3K-Akt pathway components were significantly upregulated in intestinal co-cultures (Fig. S2C), and fibroblasts derived from intestinal and pulmonary co-cultures clustered separately in PCA analysis and volcano plot (Fig. 2B, Fig. S2B), indicating transcriptional divergence.

### Single-cell deconvolution

**Fig. 4:**
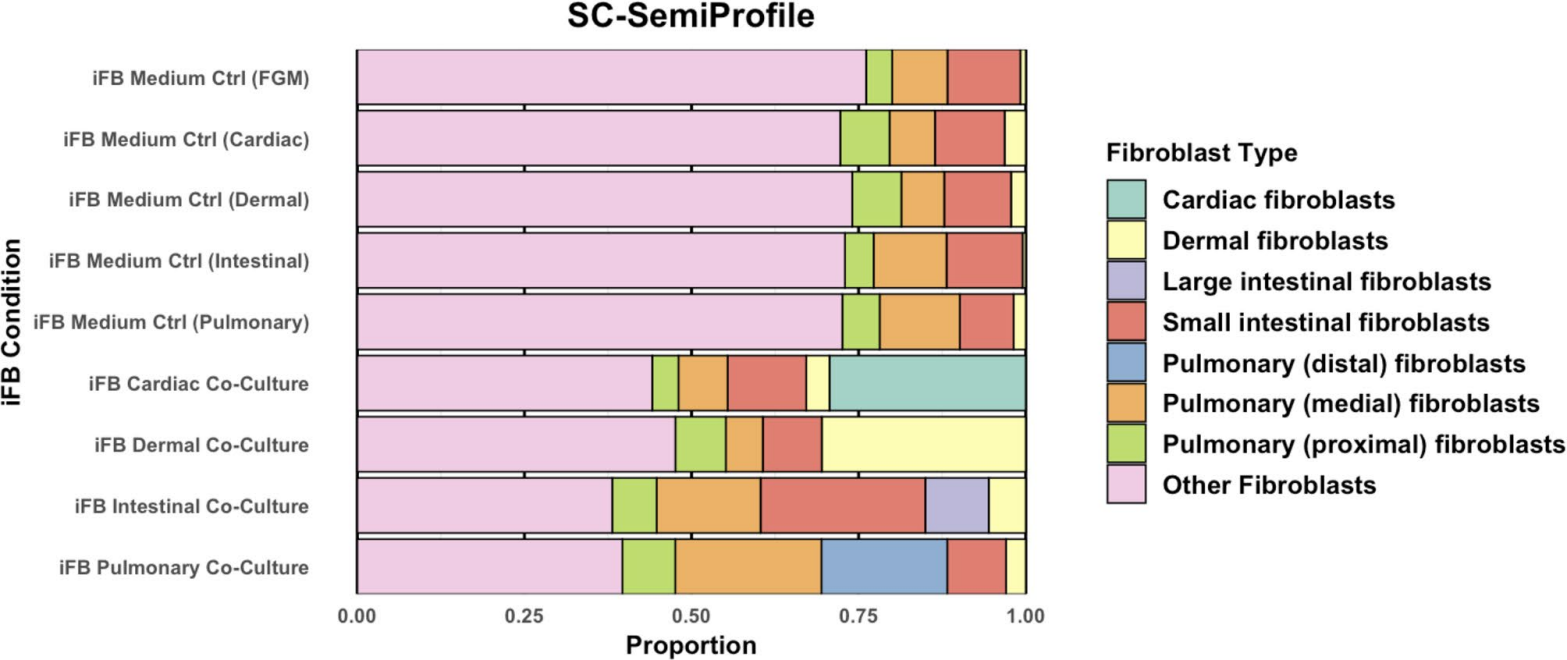
The stacked bar plot represents the proportional distribution of fibroblast subtypes across iFB conditions, as inferred from SemiProfiler against a reference set of over 20,000 primary fibroblast single-cell RNA sequencing profiles. “Other Fibroblasts” (a heterogeneous pool spanning various organs such as liver, uterine, and mammary tissues)

To further characterise the transcriptional profiles of iFBs, we applied scSemiProfiler to deconvolute bulk RNA-seq data and infer single-cell-like transcriptomes (Fig. 4). This analysis generated inferred single-cell-like transcriptomes, which were subsequently compared to a reference atlas of over 20,000 fibroblast single-cell RNA-seq profiles from the Tabula Sapiens database [15, 16]. Across all co-culture conditions, iFBs exhibited heterogeneous transcriptional identities, comprising a mixture of fibroblast subtypes rather than converging on a uniform, tissue-specific signature.

Compared to their iFB Medium Control counterparts, direct co-culture with tissue-specific cells led to an increased representation of relevant fibroblast subtypes—cardiac, dermal, intestinal (small and large), and pulmonary (distal, medial, proximal), and a concurrent reduction in “Other” fibroblasts, defined as 21 reference fibroblast types not targeted in this study (e.g., bladder, liver, pancreas). Cardiac and dermal co-cultured iFBs adopted a tissue-specific fibroblast subtype of above 25% each after 7 days of co-culture. iFBs with intestinal co-culture displayed an increased proportion of both large (purple) and small (red) intestinal fibroblasts, without appearing to distinctly favour one over another. Large intestinal fibroblasts and pulmonary distal fibroblasts only appeared visibly present in their respective iFB intestinal and pulmonary co-cultures. All iFB populations retained mixed transcriptomic contributions from both targeted and non-targeted fibroblast lineages.

### Direct vs. indirect co-culture

**Fig. 5:**
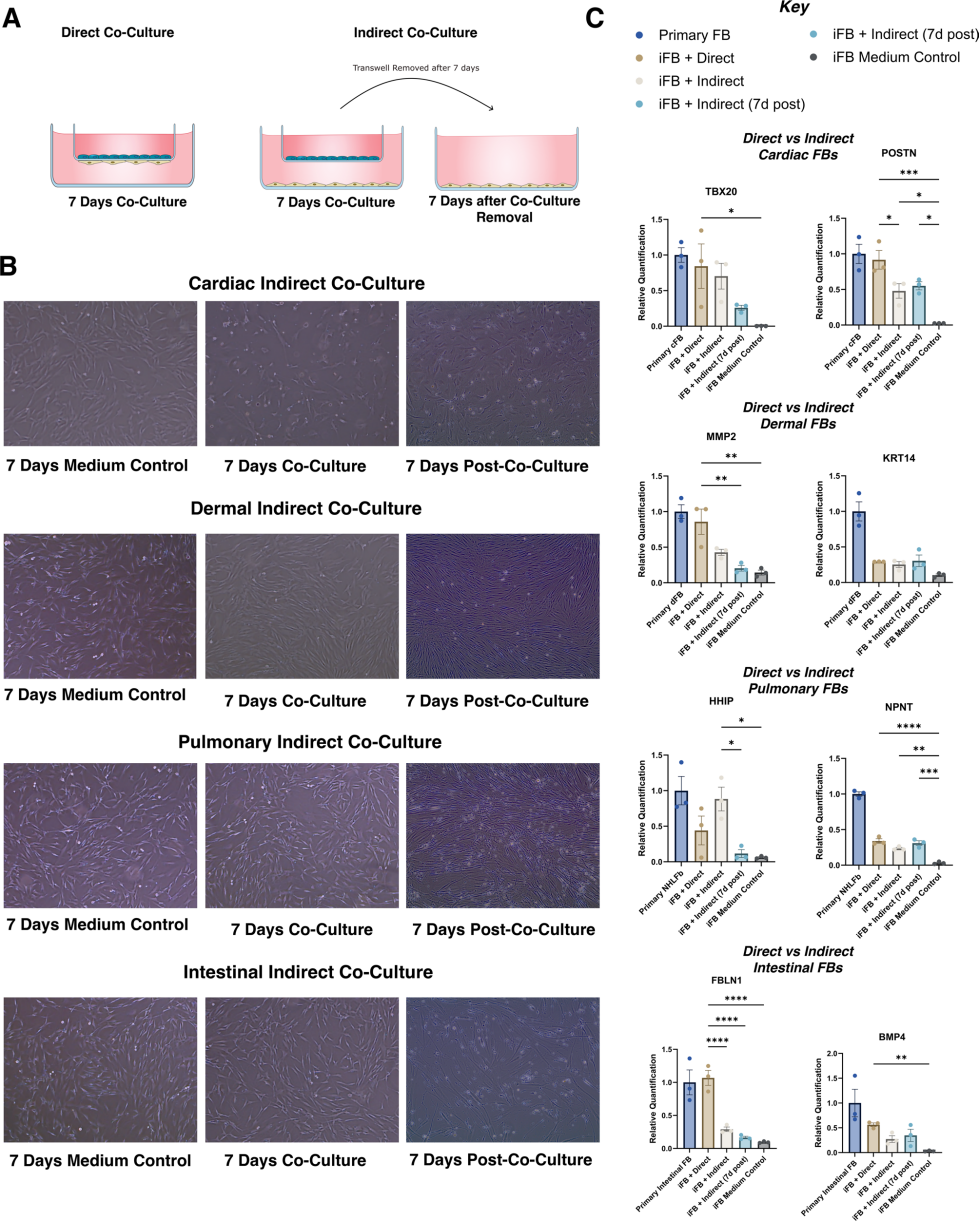
(**A**) Scheme of direct and indirect co-culture systems, illustrating the transwell configuration for indirect co-culture. (**B**) Representative bright-field images of iFBs after 7 days of indirect co-culture and 7 days post-co-culture with cardiac, dermal, pulmonary, and intestinal cells. (**C**) Transcript expression of organ-specific markers measured via qPCR. “Indirect” refers to transwell co-culture conditions where iFBs were continuously exposed to paracrine signals. “Indirect (removed)” indicates iFBs that were co-cultured indirectly, then removed from co-culture and cultured alone for an additional 7 days prior to analysis. Abbreviations: NHLFb– normal human lung fibroblast; cFB– cardiac fibroblast; dFB– dermal fibroblast. Data are presented as mean ± SEM, *n* = 3 cell lines per condition. One-way ANOVA was performed, *P <.05, **P <.01, ***P <.001, P****<0.0001

In indirect co-culture systems utilising transwell inserts to physically separate iFBs from organ-specific cells (Fig. 5A), we observed persistent morphological adaptations in cardiac and dermal iFBs when comparing co-cultured versus medium-only controls (Fig. 5B). These changes were accompanied by detectable transcriptional shifts, with some changes substantially attenuated compared to direct co-culture conditions where iFBs interact with neighbouring cells across a shared transwell membrane (Fig. 5C).

For instance, in direct co-culture, *MMP2* and *FBLN1* transcript levels approached those of primary fibroblasts (*MMP2* fold-change = 0.86, *FBLN1* fold-change = 1.06, relative to primary fibroblasts), whereas in indirect co-culture they remained lower (*MMP2* fold-change = 0.42, *FBLN1* fold-change = 0.28, relative to primary fibroblasts). Markers such as *POSTN*, *TBX20*, and *HHIP* however retained elevated expression under indirect conditions relative to isolated iFBs without co-culture.

Some transcriptional responses to indirect co-culture were not maintained upon removal of the paracrine stimulus. For example, *TBX20* expression declined from a fold-change of 0.77 during co-culture to 0.26 post-removal, suggesting transient effects. Morphological changes were more pronounced between the different media controls than between the culture conditions (Fig. 5B*)*.

## Discussion

This study set out to determine whether iFBs can acquire transcriptional tissue-specific traits through co-culture with epithelial and mesoderm cells from different organs. Using a transwell-based co-culture system, we explored the transcriptional plasticity of iFBs with the ultimate aim of assessing their suitability for integration into complex, human-based models of high biomimicry. Our data demonstrate that iFBs exhibit notable transcriptional plasticity in response to co-culture with cells from all three distinct germ layers, clustering alongside their primary fibroblast counterparts in the PCA heatmap. This responsiveness was consistent across multiple iPSC lines, suggesting that co-culture indeed contextually tune iFBs, potentially bypassing the need for lineage-specific differentiation protocols. Although fibroblast plasticity is well-established [17, 18] this finding demonstrates that iFBs acquire transcriptional traits of mesodermal, ectodermal, and endodermal fibroblasts when exposed to diverse tissue-specific microenvironments.

To evaluate the fidelity of this plasticity, we assessed the expression of markers enriched in organ-resident fibroblasts. While several organ-specific markers, such as *POSTN* (cardiac FB) and *MMP2* (dermal FB), showed tissue-paired upregulation, others, including *BMP4* and *FBLN1*, were induced across multiple co-culture conditions, despite their intestinal fibroblast associations [19, 20]. This pattern may reflect either shared fibroblast programs or generalised responses to juxtacrine/paracrine signals [3, 20]. These observations are consistent with the known limitations of classical fibroblast markers [20] which often lack strict tissue exclusivity due to overlapping roles in ECM remodelling, wound repair, and inflammation. For instance, the widespread induction of *BMP4* across conditions, despite its role in intestinal mesenchyme development [19] points to a conserved epithelial-mesenchymal signalling that transcends tissue boundaries. Collectively, these findings emphasise iFB flexibility in diverse microenvironments and highlight the ongoing challenge of defining truly lineage-restricted fibroblast markers.

Building on these observations, we investigated whether iFB transcriptional plasticity translates into pathway-level differences across co-culture conditions. Fibroblasts behaviour is finely tuned to their organ of origin and varies considerably across both organ-specific and intra-organ subtypes [3, 21]. In cardiac co-cultures, for example, genes in the TGF-β pathway were upregulated, in line with the central role of cardiac fibroblasts in myocardial repair [22] alongside elevated calcium signalling genes supporting cardiomyocyte electrophysiology and excitation-contraction coupling [23].

In contrast, iFBs cultured in non-cardiac environments exhibited distinct and sometimes opposing transcriptional programmes. Wnt signalling was modulated in a context-dependent manner: cardiac co-cultured iFBs upregulated *WNT7A*, *LGR5*, and *SOX17*, genes associated with cardiac development and regeneration [24–26] whereas downregulated Wnt-related components in dermal iFBs may be indicative of a quiescent fibroblast state focussing on skin barrier maintenance over active regeneration [27, 28]. Dermal iFBs also displayed broad expression in the ECM receptor interaction pathway, underscoring the complex and diverse roles of dermal fibroblasts in ECM remodelling across fibroblast populations in the skin [29].

Pulmonary iFB co-cultures presented a more complex picture. iFBs co-cultured with NHBEs clustered with primary lung fibroblasts at a global level (Fig. 2B, Fig. S4), but failed to activate canonical lung pathways such as TGF-β/Smad [30], suggesting that tissue-specific traits may be underrepresented in our setup. Interestingly, despite their shared endodermal origin, intestinal and pulmonary iFBs exhibited divergent pathway activation profiles. This divergence likely reflects organ-specific physiological demands: lung fibroblasts prioritise structural maintenance and immune modulation to sustain barrier integrity in a relatively low-turnover environment, whereas intestinal fibroblasts promote PI3K-Akt pathway activity to support rapid epithelial renewal and mucosal repair in the intestine’s high-renewal niche [31].

The modest pathway enrichment across co-culture conditions may reflect a relatively quiescent iFBs state, absent of activating cues. For example, pulmonary fibrosis-associated markers like *CTHRC1* were not induced [32] suggesting that pro-inflammatory, mechanical or injury-related cues are necessary to reveal key tissue-specific features.

To reconcile global and pathway-level discrepancies, we applied scSemiProfiler to deconvolute bulk RNA-seq data against Tabula Sapiens fibroblast references. Rather than adopting uniform organ-specific fibroblast signatures, iFBs showed mixed identities, enriched for tissue-relevant but not exclusive fibroblast signatures. Co-culture reduced this heterogeneity, but did not eliminate it, suggesting that this residual diversity may reflect aspects of physiological fibroblast diversity both across and within tissues. For instance, intestinal co-cultures included fibroblasts resembling both small and large intestine, even when biased toward a small intestine (jejunum). This aligns with prior observations that individual organs can harbour multiple resident fibroblast subtypes [3, 20].

The persistent heterogeneity likely stems from both biological and technical constraints that limit full lineage commitment. Factors including epigenetic inertia, inter-line iPSC variability, limited culture duration and suboptimal media, likely impede complete lineage specification. Moreover, it should be noted that while our direct co-culture facilitates close cellular communication, the presence of the transwell membrane likely limits classical juxtacrine signalling. Given that fibroblast identity exists along a continuum [17, 18] achieving fully functional, tissue-specific populations will require more complex environmental cues, potentially involving matrices, mechanical inputs, or extended co-culture durations. In addition to the need for broader marker panels, functional assays (e.g., ECM composition) will also be needed to accurately assess inter- and intra-organ fibroblast identity and function.

While iFBs exhibit transcriptional plasticity in response to their environment, it remained unclear whether these changes represent stable or transient lineage adaptions. To address this, we tested whether a paracrine-only indirect co-culture setup could induce and sustain tissue-specific gene expression. iFBs are often utilised in either direct 2D or 3D co-cultures that enable cell-cell contact and better mimic in vivo conditions [6, 33] or in indirect co-cultures where physical separation between fibroblasts and other cells facilitates cell-type specific downstream analyses such as immunofluorescence or Western blot [34, 35].

Our data indicate that although markers like *POSTN*, *TBX20*, and *HHIP* remain elevated in indirect co-cultures relative to isolated medium-only iFBs, the transcriptional effects of indirect co-culture were generally less pronounced compared to direct co-cultures. Moreover, contrary to findings in primary mouse fibroblasts [36] many transcriptional alterations in iFBs diminish after stimulus removal (e.g. *TBX20* fold-change with indirect co-culture = 0.77, *TBX20* fold-change after co-culture removal = 0.26), implying that continuous paracrine signalling is required to sustain tissue-specific gene expression.

We also observed media-dependent morphological variation, suggesting that media composition exert a strong influence on fibroblast morphology and possibly molecular state, though without affecting iFB identity (Figs. 2B and 4; Fig. S2). Together, these findings underscore the dynamic plasticity of iFBs and suggest that achieving stable, tissue-specific fibroblast phenotypes will likely depend on direct cell-cell contact and prolonged environmental cues. This is an important consideration for modelling chronic diseases such as fibrosis and wound healing, where long-term maintenance of fibroblast identity is essential.

## Conclusion

Developing human-based model systems remains essential for capturing the physiological relevance of human tissues and enabling the study of tissue repair, immune modulation, fibrosis and other context-dependent fibroblast behaviours. We demonstrate that iFBs can partially adopt tissue-specific transcriptional profiles matching primary cardiac, dermal, pulmonary and intestinal fibroblasts, with pathway analyses and single-cell deconvolution revealing incomplete specification. While paracrine signalling cues induce transient changes, highlighting iFB plasticity, sustained interactions appear necessary for stable phenotypes. These findings underscore the need to validate functional ECM remodelling and contractile capacities in 3D or dynamic models to confirm their relevant use in complex tissue models. Additionally, future studies should also explore whether iFBs display enhanced regenerative or therapeutic potential in vivo, for example through functional assays such as wound healing models. Crucially, our single differentiation protocol yields iFBs adaptable across multiple organ contexts, providing a practical platform for patient-specific disease modelling without the need for multiple lineage-specific methods. Finally, although iFBs have been regularly incorporated into multicellular systems, to our knowledge this is the first demonstration that they transcriptionally adapt to distinct microenvironments in a context-dependent manner.

## Electronic supplementary material

Below is the link to the electronic supplementary material.

Supplementary Material 1

Supplementary Material 2

Supplementary Material 3

Supplementary Material 4

Supplementary Material 5

Supplementary Material 6

Supplementary Material 7

## Data Availability

The datasets during and/or analysed during the current study available from the corresponding author on reasonable request. The bulk RNA sequencing data can be found on GEO (https://www.ncbi.nlm.nih.gov/geo/query/acc.cgi?acc=GSE294057). Publicly archived datasets, including Tabula Sapiens, were used and are available on Figshare. Annotated and partially annotated objects, compatible with Scanpy and Chan Zuckerberg CELLxGENE, can be accessed from Figshare at https://figshare.com/articles/dataset/Tabula_Sapiens_v2/27921984.
