# Supplementary material for: Unveiling tissue-specific transcriptional adaptations in iPSC-derived fibroblasts via co-culture systems
Source: Stem Cell Res Ther. 2025 Jul 30;16:413. doi: 10.1186/s13287-025-04537-6 (PMC12312452; doi:10.1186/s13287-025-04537-6)
Supplement: Supplementary file 1 — Supplementary Material 1 [file 13287_2025_4537_MOESM1_ESM.pdf]

SUPPLEMENTARY INFORMATION

Unveiling Tissue-Specific Transcriptional Adaptations in iPSC-Derived Fibroblasts via Co-Culture Systems

Amar J. Azad[1], Alessandro Bentivogli[1], Henrike Germar[1], Dana Wörz[1], Elena Lizunova[1], Max J. Cumberland[2], January Weiner 3rd [1], Sarah Hedtrich[1,3,4,5]

[1] Berlin Institute of Health at Charité, Berlin, Germany

[2] The Westmead Institute of Medical Research, Westmead, New South Wales, Australia

[3] School of Biomedical Engineering, The University of British Columbia, Vancouver, Canada

[4] Faculty of Pharmaceutical Sciences, The University of British Columbia, Vancouver, BC, Canada

[5] Centre for Blood Research & Life Science Institute, University of British Columbia, Life Sciences Centre, Vancouver, British Columbia, Canada.

Corresponding Authors: Dr. Amar Azad, Current Address: Berlin Institute of Health at Charité, *Käthe-Beutler-Haus,* Berlin, Germany. Email: amar_azad@outlook.com.

Prof. Dr. Sarah Hedtrich, Current Address: School of Biomedical Engineering, The University of British Columbia; 2350 Health Sciences Mall, Vancouver, BC V6T 1Z3, Canada. Email: sarah.hedtrich@ubc.ca; phone: +1-604-822-2466.

Supplementary Table 1. Primer List

Name	Sequence
TBX20 FWD	ggCgACggAgAACACAATCAA
TBX20 REV	CTgggCACAggACgACTTC
MMP2 FWD	TACAggATCATTggCTACACACC
MMP2 REV	ggTCACATCgCTCCAgACT
HHIP FWD	TCTCAAAgCCTgTTCCACTCA
HHIP REV	gCCTCggCAAgTgTAAAAgAA
NPNT FWD	gTAAgCACAggTgCATgAACA
NPNT REV	gAACCATCCggCATgAgCATA
BMP4 FWD	ATgATTCCTggTAACCgAATgC
BMP4 REV	CCCCgTCTCAggTATCAAACT
CXCL12 FWD	ATTCTCAACACTCCAAACTgTgC
CXCL12 REV	ACTTTAgCTTCgggTCAATgC
FBLN1 FWD	AgAgCTgCgAgTACAgCCT
FBLN1 REV	CgACATCCAAATCTCCggTCT
POSTN FWD	CACgCAAgCCATTATCTCTCCA
POSTN REV	ACCCACTCATATAgAAATgTgCAAAgC
KRT14 FWD	ggCCTgCTgAgATCAAAgACTAC
KRT14 REV	CACTgTggCTgTgAgAATCTTgTT
18S FWD	CGCGGTTCTATTTTGTTGGT
18S REV	AGTCGGCATCGTTTATGGTC

Supplemental Figures

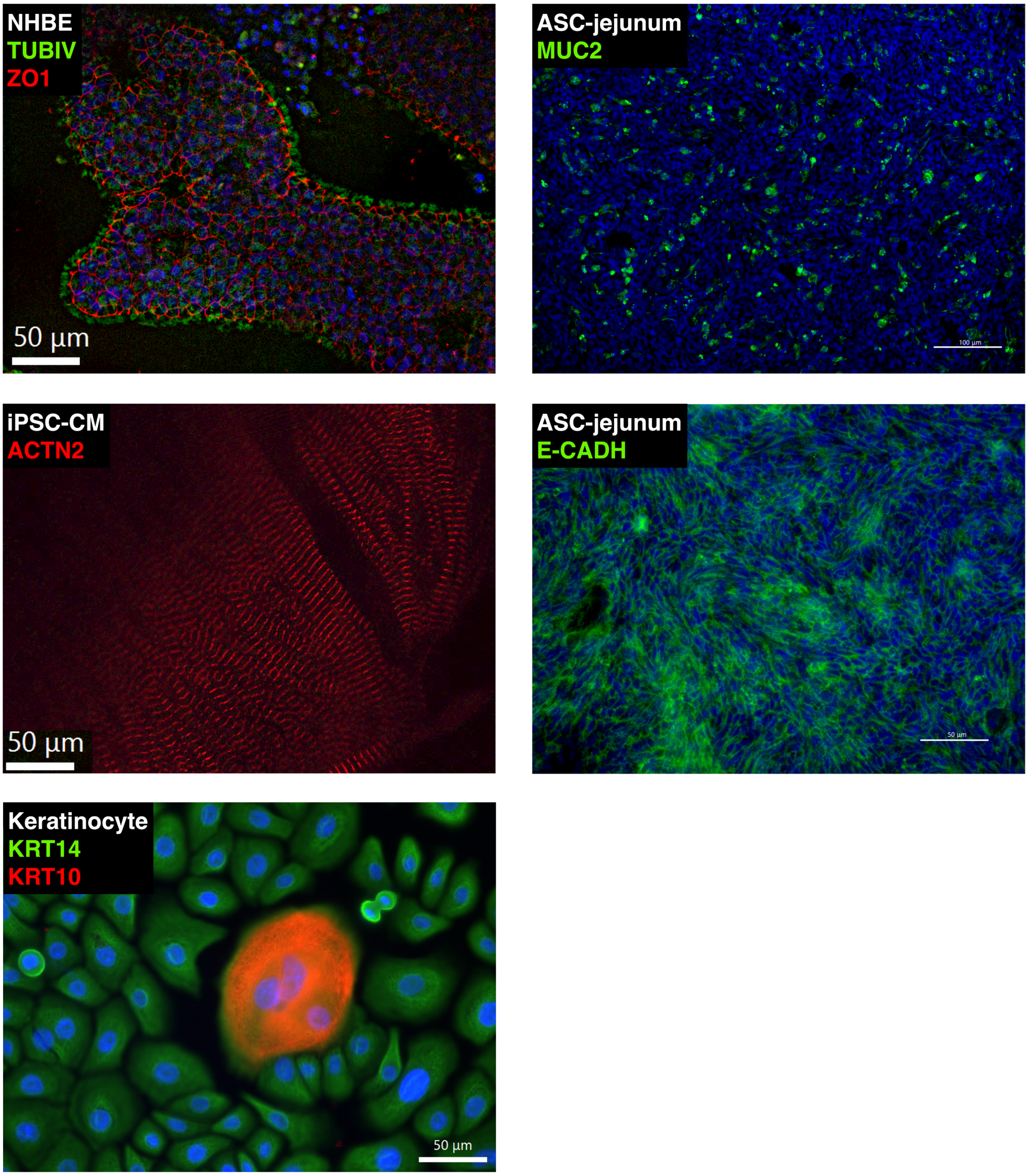

Supplementary Figure 1 Immunofluorescence characterisation of NHBE, iPSC-CM, ASC-derived jejunum and primary keratinocytes.

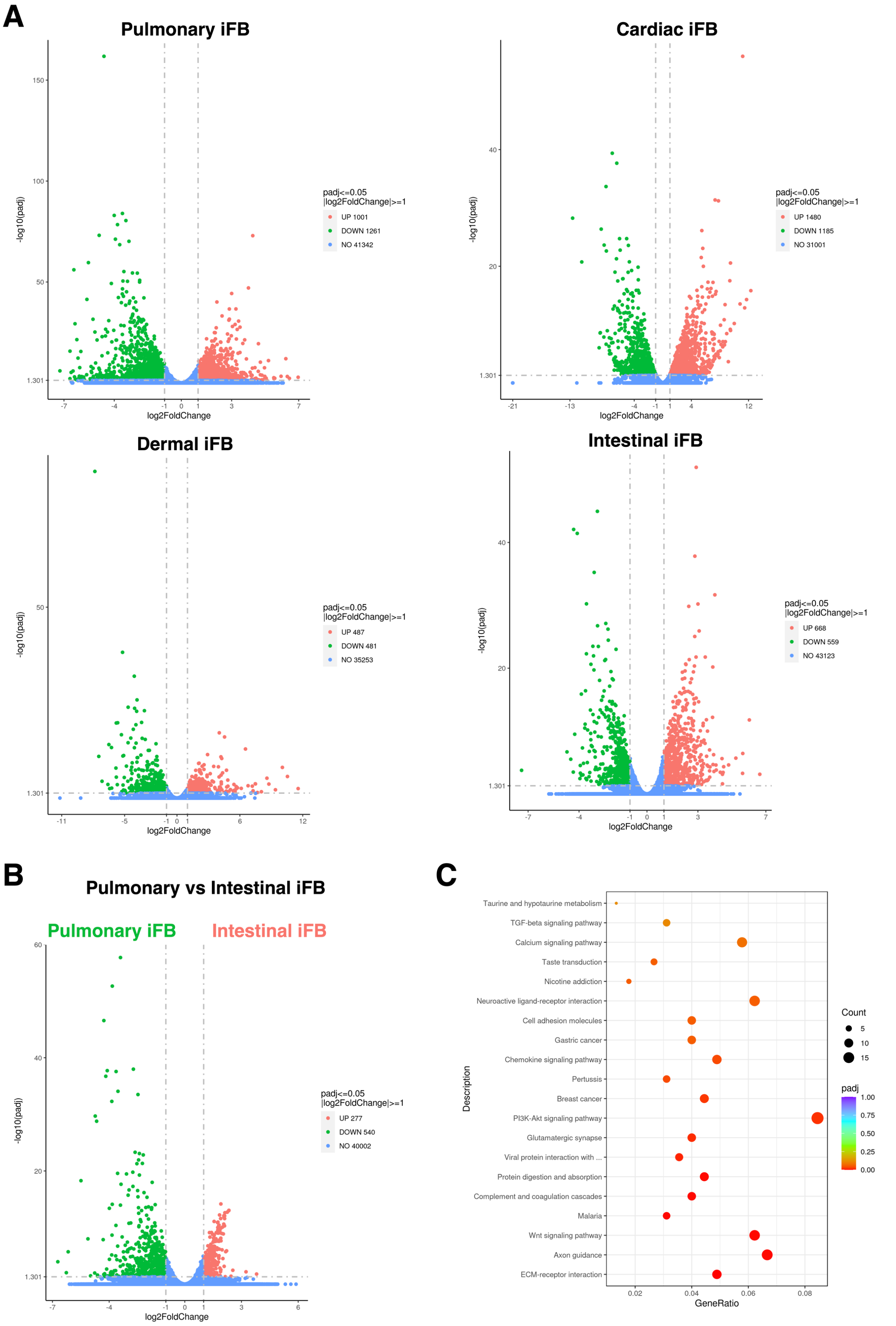

Supplementary Figure 2 (A) Volcano plots of co-cultured iFBs compared to non-co-cultured iFBs in the basal medium for co-culture. (B) Volcano plot comparing iFBs co-cultured with NHBEs with ASC-jejunal cells. (C) Dot plot depicting significantly upregulated KEGG pathways in ASC-jejunal-co-cultured iFBs compared to NHBE-co-cultured iFBs.

A

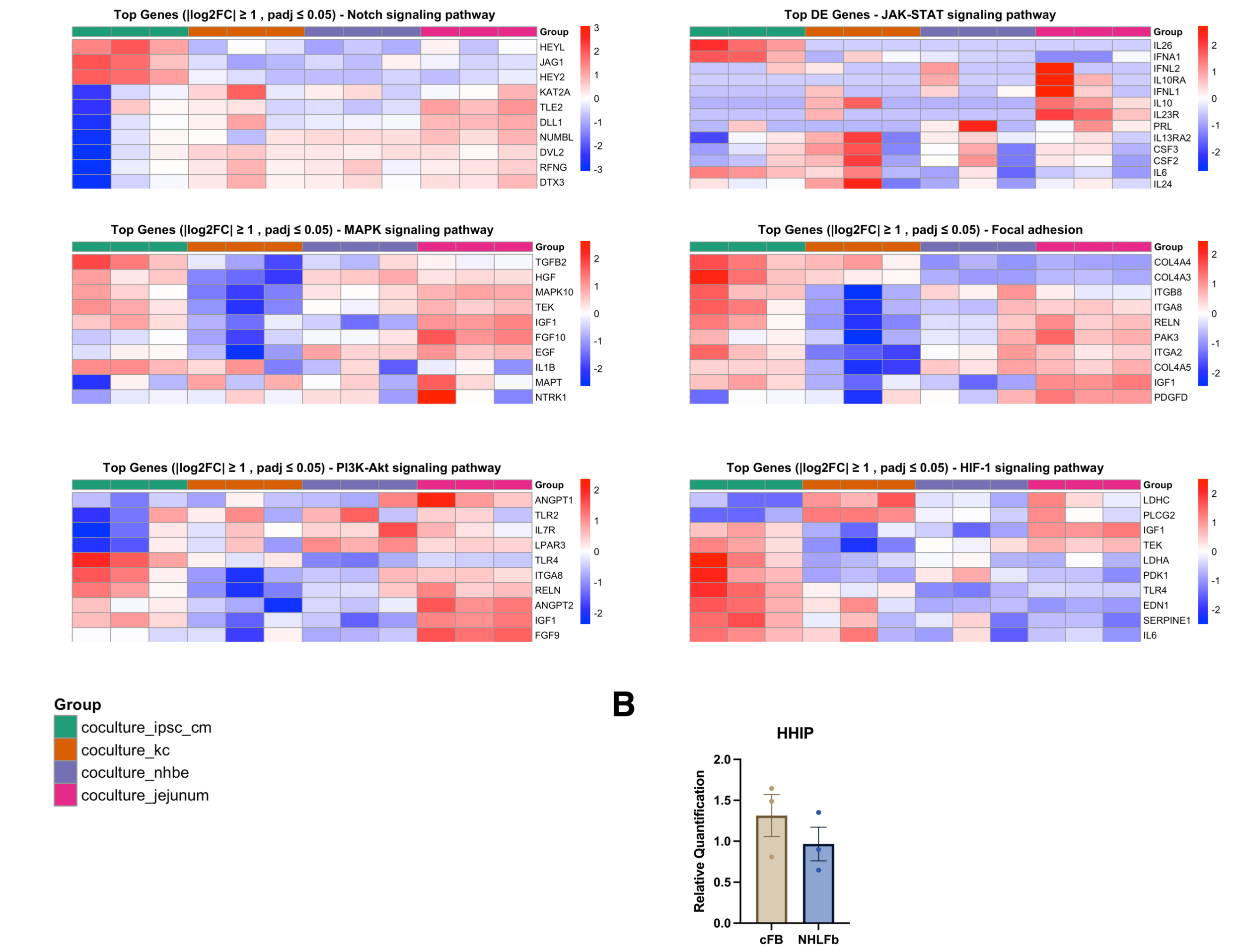

Supplementary Figure 3 (A) Heatmaps depicting the top 5 differentially expressed (DE) genes across key KEGG pathways. (B) HHIP transcript expression in primary cardiac fibroblasts (cFB) and normal human lung fibroblasts (NHLFb).

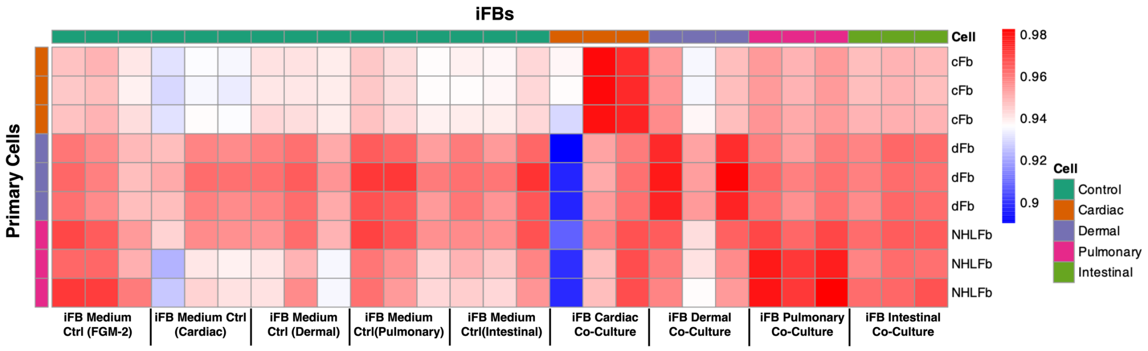

Supplementary Figure 4 Expression correlation heatmap comparing RNA-seq datasets of different culturing conditions of iFBs with cardiac (cFB), dermal (dFB), and normal human lung fibroblasts (NHLFs), demonstrating transcriptional overlap between iFBs with primary fibroblasts.
