# Supplementary figures and images for: Unveiling tissue-specific transcriptional adaptations in iPSC-derived fibroblasts via co-culture systems
Source: Stem Cell Res Ther. 2025 Jul 30;16:413. doi: 10.1186/s13287-025-04537-6 (PMC12312452; doi:10.1186/s13287-025-04537-6)

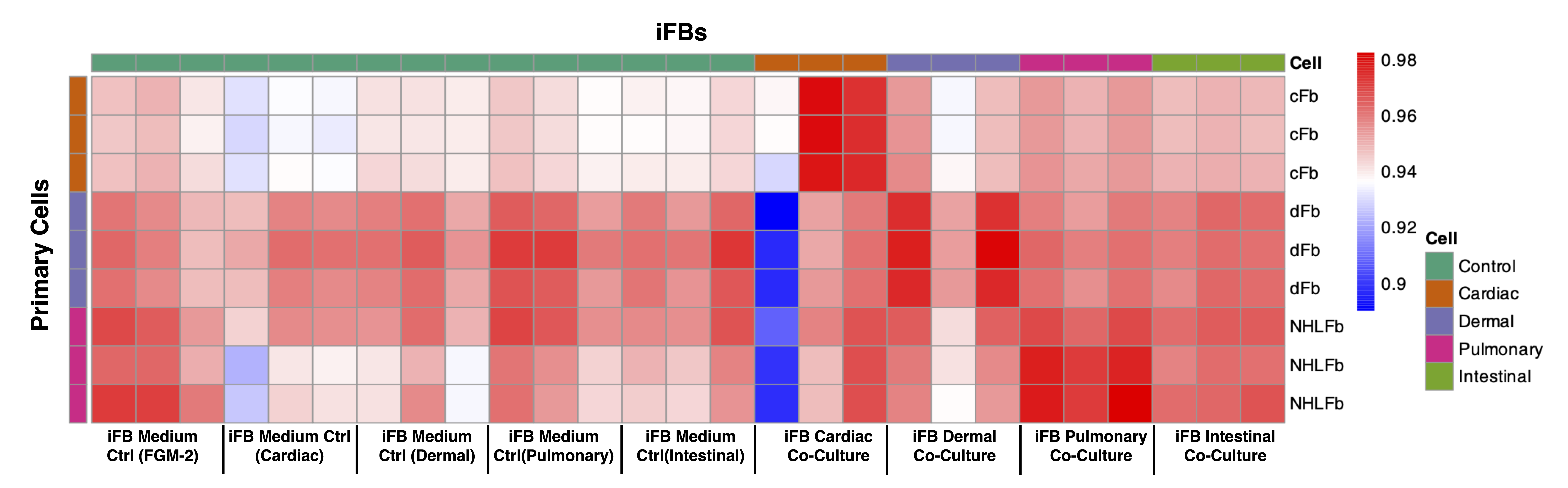

Supplement: Supplementary file 5 — Supplementary Material 5 [file 13287_2025_4537_MOESM5_ESM.png]
